# Association of Different Forms of Child Maltreatment With Peer Victimization in Mexican Children and Adolescents

**DOI:** 10.3389/fpsyg.2021.662121

**Published:** 2021-08-06

**Authors:** Javier Martín-Babarro, M. Paz Toldos, Lorena Paredes-Becerra, Renzo Abregu-Crespo, Juan Fernández-Sánchez, Covadonga M. Díaz-Caneja

**Affiliations:** ^1^Departamento de Investigación y Psicología en Educación, Universidad Complutense de Madrid, Madrid, Spain; ^2^Tecnologico de Monterrey, Monterrey, Mexico; ^3^Department of Child and Adolescent Psychiatry, Institute of Psychiatry and Mental Health, Hospital General Universitario Gregorio Marañón, IiSGM, CIBERSAM, School of Medicine, Universidad Complutense, Madrid, Spain; ^4^School of Psychology, Universidad Nacional de Educación a Distancia, Madrid, Spain

**Keywords:** peer victimization, bullying, child malteratment, adolescents, development, polyvictimization

## Abstract

**Objective:** To examine the relationship between exposure to multiple forms of child abuse and neglect within the family context and peer victimization at school, accounting for the moderator effect of sex and educational level.

**Methods:** Two thousand four hundred fifteen children and adolescents, aged 9 to 15 years, attending public schools in Mexico completed the Childhood Trauma Questionnaire-Short Form and a modified version of the Olweus' Bully/Victim Questionnaire. We used linear regression models to assess the association of five different forms of child abuse (emotional, physical, and sexual abuse, and emotional and physical negligence) with three forms of peer victimization (direct, indirect, and cyberbullying).

**Results:** Direct forms of child abuse within the family (i.e., emotional, physical, and sexual abuse), but not neglect, were significantly and positively associated with a risk for peer victimization. In the fully adjusted models, emotional abuse was significantly associated with the three types of peer victimization: [indirect *b* = 0.48, *t* = 6.75, *p* < 0.001, direct (*b* = 0.47, *t* = 4.89, *p* < 0.001), and cyberbullying (*b* = 0.85, *t* = 5.45, *p* < 0. 001)]; while physical abuse was positive and significantly associated with direct victimization (*b* = 0.29, *t* = 3.28, *p* < 0.001). Boys suffering from sexual abuse within the family context showed higher levels of all subtypes of peer victimization. Students attending secondary school who suffered from sexual abuse showed higher levels of indirect victimization than did students attending primary schools.

**Conclusion:** Child abuse within the family context seems to be associated with the risk of peer victimization. Preventive strategies to address bullying and promote resilience should take family factors into account. Interventions for high-risk families might be useful to prevent child multi-victimization.

## Introduction

Increasing evidence gathered in the past few decades supports the need to adopt a preventive approach to psychiatry (OECD, 2011; Sommer et al., 2016). Risk and protective factors acting during sensitive stages of neurodevelopment such as pregnancy, childhood, and adolescence, have a long-term impact on mental health across the lifespan, which suggests that primary preventive strategies should be implemented during the earlier stages of development (Parellada, 2013). Potentially preventable risk factors during childhood and adolescence include exposure to violence within the family and school contexts, including child abuse and peer victimization or bullying (Moreno-Peral et al., 2017). Both factors have been associated with negative short- and long-term psychiatric, educational, and medical outcomes and with increased risk of suicide (Green et al., 2010; Takizawa et al., 2014).

Bullying can be defined as a subtype of aggression among peers, characterized by the display of intentioned, repetitive, and negative actions (physical, verbal, relational aggression, including using online media) in the context of an imbalance of power between victim and aggressor (Olweus, 1993; Williams and Guerra, 2007). The American Psychological Association (2004) deemed it a major public health concern, with a mean prevalence of more than 35% for traditional bullying and 15% for cyberbullying (Modecki et al., 2014). Castellvi et al. (2017) estimated that more than one fifth of completed suicides before age 26 could be prevented by suppressing all forms of bullying.

Several scholars have explored the effect of family variables on bullying, such as hierarchical structures, parenting strategies, parental warmth, or intra-parental violence (Bowers et al., 1992; Dodge et al., 1997; Baldry, 2003; Gershoff et al., 2010; Hong et al., 2012). Although previous evidence suggests that exposure to different forms of violence during childhood and adolescence might be interrelated, and thereby increasing the risk of repeated victimization (Finkelhor et al., 2015), specific research on the potential association of child abuse within the family with the risk of peer victimization is still scarce (Duncan, 1999; Dussich and Maekoya, 2007). Longitudinal studies suggest that child emotional and physical maltreatment is associated with increased peer rejection, and that this association might be mediated by emotional dysregulation, externalizing symptoms, or increased aggressiveness (Bolger and Patterson, 2001; Kim and Cicchetti, 2010). Other studies have reported a positive association of physical abuse, sexual abuse, and negligence with an increased risk of peer rejection or bullying (Bolger and Patterson, 2001; Shields and Cicchetti, 2001; Dussich and Maekoya, 2007). This association can be understood in the context of ecological-transactional models (Cicchetti and Lynch, 1993), which assume that individual development is shaped by the multiple interactions and transactions of risk and protective factors between nested levels of influence (family, school, peers, and media) (Cicchetti and Rizley, 1981; Espelage and Swearer, 2003; Hong and Espelage, 2012; Hong et al., 2012; Petersen et al., 2014). These models have tried to guide the conceptualization of child abuse (Petersen et al., 2014) and the various forms of peer victimization (Espelage and Swearer, 2003; Hong and Espelage, 2012). Within this theoretical model, both social phenomena would be the product of a series of complex interactions between intra- and inter-individual variables (Espelage and Swearer, 2003). A child's individual characteristics interact with family variables, which in turn are embedded in a broader social ecological system, including communities, neighborhoods, and other cultures. There is also a consensus in considering that the relationship between child abuse and the subsequent development of peer victimization is due to multi-causality, and that the characteristics of different social contexts in which children and adolescents interact mediate and influence the individual characteristics, such as aggressiveness (Bronfenbrenner, 1994).

In this framework, it seems pertinent to ask about the possible effect of variables such as sex and educational level, as moderating variables, on child abuse and peer victimization, given the scarcity of studies in this regard (Swearer et al., 2010; Guerra et al., 2011). Sex appears to have a certain effect on the diverse dynamics of peer violence, including bullying (Cook et al., 2010). In general, boys seem to be more involved than girls in bullying dynamics as aggressors (Nansel et al., 2001; Carbone-Lopez et al., 2010; Cook et al., 2010; Guerra et al., 2011), and are at greater risk of direct forms of bullying. On the other hand, girls tend to be equally or more likely to experience indirect forms of bullying (e.g., Rivers and Smith, 1994; Baldry and Farrington, 1998; Putallaz et al., 2007). However, not all studies confirm sex as a moderating variable between child abuse and peer victimization (Shields and Cicchetti, 2001). Age, and therefore educational level, also has a moderating effect on the dynamics of peer victimization. In general, peer victimization tends to increase during childhood, peaking during early adolescence and declining during adolescence (Nansel et al., 2001). Williams and Guerra (2007) have shown that both physical peer victimization and cyberbullying peak during the last years of primary education and then decline during secondary education, while verbal bullying reaches its peak at the end of primary education and remains relatively stable during secondary education. Cook et al. (2010), in a recent meta-analysis, examined differences based on sex and educational level and concluded that the relationship between internalization and peer victimization becomes stronger over time.

In this study, we aimed to assess the association of different forms of child maltreatment (physical, sexual, and emotional abuse, as well as emotional and physical neglect) within the family context with different categories of peer victimization within the school context (indirect, direct, and cyberbullying) in a large sample of Mexican students. Considering recent evidence that physical, sexual, and emotional abuse and various forms of neglect of children are associated with substantially increased risk of concurrent and subsequence psychopathology based on sex or due to developmental differences (Zeanah and Humphreys, 2018), we also explored the potential moderating effect of sex and educational level on this association. To our knowledge, only Kim and Cicchetti (2010) have assessed the effect of different forms of maltreatment on difficulties with peers. In this study, they reported that both physical and sexual abuse, as well as neglect were associated with increased rates of peer rejection. And in Mexico, we found no previous studies that specifically assessed the differential effect of multiple forms of maltreatment on different subtypes of peer victimization or explored the effect of sex or educational level on the association between specific forms of child abuse and of peer victimization.

Previous scholars have reported that the direct forms of abuse within the family context (e.g., physical and emotional abuse) are associated with increased peer victimization (Duncan, 1999) and perpetration (Shields and Cicchetti, 2001). We hypothesized that those forms of child maltreatment in which the aggressor exerts direct abuse (i.e., emotional, physical, or sexual abuse), would be positively associated with a greater peer victimization compared to those passive or indirect forms of child maltreatment based on neglecting strategies. Additionally, some authors have claimed that sex and educational level moderate the relationship between child abuse and peer victimization, such that boys are at greater risk of direct forms of bullying and child maltreatment (Nansel et al., 2001; McCarroll et al., 2008; Carbone-Lopez et al., 2010; Cook et al., 2010; Guerra et al., 2011), and peer victimization becomes stronger over time; therefore, we hypothesized that sex and educational level would moderate the relationship between child abuse and peer victimization, such that boys would report higher peer bullying when exposed to direct forms of abuse at home.

## Methods

### Procedure and Participants

This research was performed within the framework of “Prevention of family violence,” a program for teachers, school officials, and pedagogical technical advisors for elementary and secondary education, organized and conducted by “Educadores sin Fronteras,” (Teachers without Borders) a non-governmental organization (NGO), in collaboration with the Sindicato Nacional de Trabajadores de la Educación (SNTE) (National Syndication of Education Workers). We invited all program participants to participate voluntarily in this research, and we requested the pertinent permissions from each of the centers that agreed to collaborate. A total of 73 public schools from 19 states out of the total 32 of the Republic of Mexico participated in this research (17.2% of the participants came from schools in the northern regions of the country, 12.3% from the central region, 53.2% of the western region, and 17.3% of the southern region of the country). In addition, through a non-random sampling we assessed 2,415 students (age 9–15 years, 52.5% girls) attending compulsory education (primary or secondary education) during one regular class hour.

We collected self-reported measures in the computer rooms of each center through two different procedures: online, using Qualtrics (38.8%), and using paper and pencil questionnaires (61.2%). We found no significant effect of the mode of completion on the scores obtained in child abuse and peer victimization measures. We obtained permission to test the students from teachers, parents, and the pertinent authorities at each school. The study complied with the ethical guidelines required for informed consent by parents, protection of personal data, and guarantees of confidentiality. In addition, we adopted ethical measures on psychological research carried out through the Internet. We informed the students that the questionnaire would be anonymous and that they could decline to answer any questions. The research team offered general information on the project and a brief description of the definitions of the different subtypes of peer victimization to ensure an appropriate comprehension of the assessment instruments. Documentation of the study can be found at https://osf.io/uq8c7/.

### Measures

#### Child Abuse and Neglect

We used the Childhood Trauma Questionnaire-Short Form (CTQ-SF) to assess different forms of abuse within the family (2003). The CTQ-SF has been widely used in trauma research in adult and pediatric samples and has been validated in different clinical populations (Kim et al., 2011; Spinhoven et al., 2014). For this study, the instrument was translated, corrected, and adapted according to the Mexican lexicon/Mexican Spanish. Moreover, to sustain the validity of the translated tool, the researchers involved in this process met the following requirements: knowledge about the concepts that the questionnaire measures; proficiency in the original language in which the instrument was written; and knowledge regarding the target population that the translation/adaptation was based on. In this way, we developed a Spanish version of the scale adapted to the Mexican population. The CTQ-SF consists of 28 items scored on a 5-point Likert scale (1 = never, 5 = very often) to assess the frequency of different situations of abuse and neglect experienced during childhood. It is composed of five subscales with high internal consistency: sexual abuse (α = 0.84); physical abuse (α = 0.78); emotional abuse (α = 0.90); physical neglect (α = 0.71); and emotional neglect (α = 0.82) (Bernstein et al., 2003; Thombs et al., 2007). Previous studies have shown high internal consistency (Bernstein et al., 2003; Gerdner and Allgulander, 2009) and test-retest reliability of the CTQ-SF (Bernstein and Fink, 1998).

#### Peer Victimization

We used a modified version of the Olweus (1996) Bully/Victim Questionnaire with 16 items to explore three dimensions of peer victimization: (1) indirect or relational, consisting of six items and defined as being victim of exclusion, rejection, or rumors (i.e., “My classmates ignore me,” “My classmates reject me”) (α = 0.80); (2) direct, composed of six items that make reference to verbal and physical victimization (i.e., “My classmates speak badly of me,” “My classmates insult, offend, or ridicule me”) (α = 0.77); and (3) cyberbullying, which consisted of four items referring to being a victim of harassment through the new technologies, such as the internet or smartphones (i.e., “My classmates speak badly of me,” “My classmates insult, offend, or ridicule me”) (α = 0.86). Participants scored each of the items based on four degrees of frequency (1 = never, 2 = sometimes, 3 = often, 4 = many times).

### Statistical Analyses

We used student *T*-tests to assess the differences in all child maltreatment and peer victimization measures by sex and educational level (secondary education (12–15 years of age) vs. primary school (grades 4–6, 9–11 years of age). We then calculated Pearson bivariate correlations among the five different types of abuse, as measured by the CTQ-SF questionnaire (emotional and physical abuse, sexual abuse, and emotional and physical neglect) and the three forms of peer victimization, as measured with Olweus bully/victim questionnaire (direct, indirect, and cyberbullying).

We used a linear regression model as the main procedure of analysis after verifying several key assumptions. Relationships among the variables were linear, and we found no multicollinearity effect after observing the correlation matrix whose coefficients presented magnitudes of 0.80 or higher. Predictor variables presented correlation values lower than 0.50. We used a Tobit model for variables that deviated from normality in the linear regression analyses (Smith and Brame, 2003). Subsequently, we performed three linear regression models, including each of the three subtypes of peer victimization as outcomes, and sex, educational level, and the five types of intrafamilial maltreatment as independent variables.

We did a Bonferroni adjustment of the *p*-value by dividing the original α-value (0.05) by the number of analyses on the dependent variable of the regression model. Finally, in order to check a possible effect of shared method variance, we followed a *post-hoc* Harman one-factor analysis to contrast variance in the data, to see if it could be largely attributed to a single factor. The percentage of variance of the sum of the squared saturations (of the extraction) was 29.32%, far less than the recommended 50%. It may be said that there was not a shared method variance influence.

## Results

Table 1 shows the mean and SD for measures of child abuse and peer victimization. Boys presented a higher level of direct victimization [*t*_(2, 413)_ = −5.43, *p* < 0.001]. No differences were found in the remaining variables related to peer victimization at school. Girls reported higher levels of emotional abuse [*t*_(2, 413)_ = 4.89, *p* < 0.001] and sexual abuse [*t*_(2, 413)_ = 2.85, *p* < 0.001]. Primary school students presented higher scores for indirect [*t*_(2, 413)_ = 4.78, *p* < 0. 001] and direct [*t*_(2, 413)_ = 2.97, *p* < 0.01] victimization, as well as for physical abuse [*t*_(2, 413)_ = 3.58, *p* < 0.001] and physical neglect [*t*_(2, 411)_ = 2.82, *p* < 0.01] than adolescents attending secondary education.

**Table 1:** Child abuse and neglect and peer victimization scores by sex and educational level.

	**Boys**	**Girls**	**Primary education**	**Secondary education**
	**(*n* = 1,151)**	**(*n* = 1,264)**	**(*n* = 858)**	**(*n* = 1,557)**
Emotional abuse	7.41 (3.31)	8.21 (4.24)	7.81 (3.53)	7.81 (4.05)
Physical abuse	6.55 (2.21)	6.38 (2.33)	6.64 (2.35)	6.30 (2.16)
Sexual abuse	5.30 (1.39)	5.49 (2.05)	5.37 (1.61)	5.41 (1.84)
Physical neglect	8.10 (3.48)	7.91 (3.44)	8.28 (3.65)	7.87 (3.37)
Emotional neglect	10.22 (4.41)	10.28 (4.66)	10.57 (4.47)	10.15 (4.54)
Indirect peer victimization	8.45 (2.90)	8.45 (2.89)	8.80 (2.92)	8.21 (2.83)
Direct peer victimization	6.89 (1.87)	6.50 (1.87)	6.80 (1.66)	6.59 (1–68)
Cyberbullying victimization	4.37 (1.12)	4.35 (1.00)	4.33 (1.00)	4.38 (1.08)

*All scores shown as mean (SD)*.

Table 2 shows the results of the bivariate correlations between measures of child abuse and peer victimization in boys and girls separately. In both sexes, all subtypes of peer victimization were significantly correlated (*r*-values ranged from 0.35 for the association between indirect victimization and cyberbullying to 0.65 for the association between direct and indirect victimization). All forms of maltreatment were also correlated, with the strongest correlation values found for the association between physical and emotional abuse (*r* = 0.55, *p*<*0.05* in boys and *r* = 0.56, *p*<*0.05* in girls). In boys, scores in all forms of child maltreatment were significantly associated with scores in the three subtypes of peer victimization (*r*-values ranging from 0.13 to 0.36), with the highest correlation values found for the association of both emotional and physical abuse with indirect victimization (*r* = 0.35, *p*<*0.05* and *r* = 0.36, *p*<*0.05*, respectively) and sexual abuse and cyberbullying (*r* = 0.33, *p*<*0.05*). In girls, scores in all forms of child maltreatment were also significantly associated with scores in the three subtypes of peer victimization (*r*-values ranging from 0.14 to 0.35), with the highest correlation values found for the association of physical abuse with both direct and indirect victimization (*r* = 0.33, *p*<*0.05* and *r* = 0.32, *p*<*0.05*, respectively) and of emotional abuse with both indirect victimization and cyberbullying (*r* = 0.41, *p*<*0.05* and *r* = 0.30, *p*<*0.05*, respectively).

**Table 2:** Correlations among the study variables for boys (below the diagonal, shaded in gray) and girls (above the diagonal).

	**1**	**2**	**3**	**4**	**5**	**6**	**7**	**8**	**9**
1. Indirect victimization	−	0.60^**^	0.39^**^	0.21^**^	0.41^**^	0.32^**^	0.18^**^	0.14^**^	−0.07^**^
2. Direct victimization	0.63^**^	−	0.56^**^	0.15^**^	0.35^**^	0.33^**^	0.21^**^	0.20^**^	−0.02
3. Cyberbullying victimization	0.35^**^	0.51^**^	−	0.12^**^	0.30^**^	0.20^**^	0.14^**^	0.22^**^	0.04
4. Emotional neglect	0.10^**^	0.16^**^	0.12^**^	−	0.36^**^	0.25^**^	0.39^**^	0.11^**^	−0.05
5. Emotional abuse	0.35^**^	0.31^**^	0.21^**^	0.25^**^	−	0.56^**^	0.25^**^	0.28^**^	0.03
6. Physical abuse	0.36^**^	0.33^**^	0.20^**^	0.17^**^	0.55^**^	−	0.21^**^	0.31^**^	−0.02
7. Physical neglect	0.13^**^	0.17^**^	0.14^**^	0.35^**^	0.26^**^	0.21^**^	−	0.20^**^	−0.07^**^
8. Sexual abuse	0.19^**^	0.29^**^	0.33^**^	0.09^**^	0.19^**^	0.16^**^	0.22^**^	−	0.04
9. Education level/school grade	−0.08^**^	−0.08^**^	0.01	−0.01	−0.05	−0.10^**^	−0.02	−0.02	−

*p < 0.05;

** *p < 0.01 (bilateral)*;

***p < 0.001.

**Figure 1:**
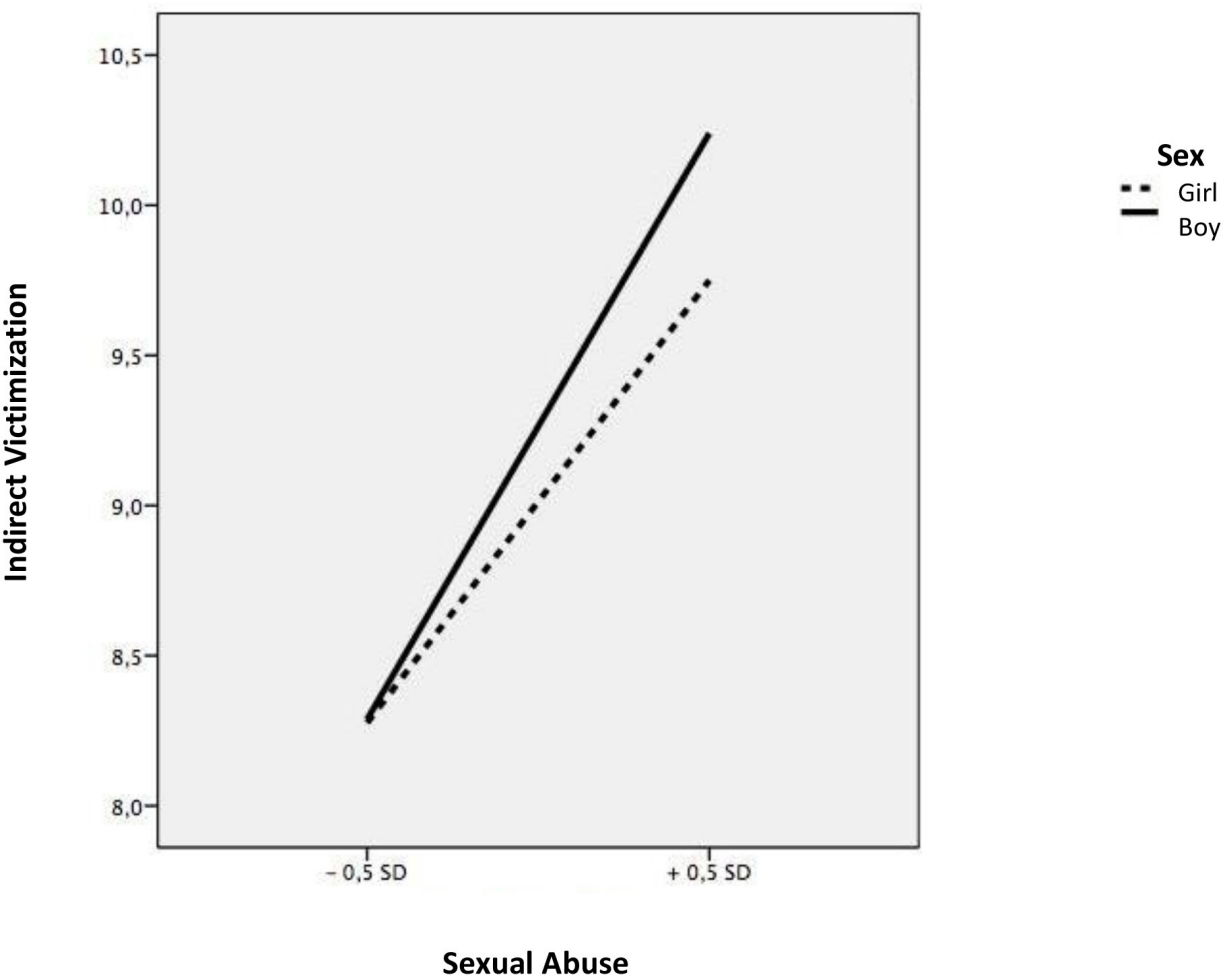
Interaction between sexual abuse and sex in relation to indirect victimization.

**Figure 2:**
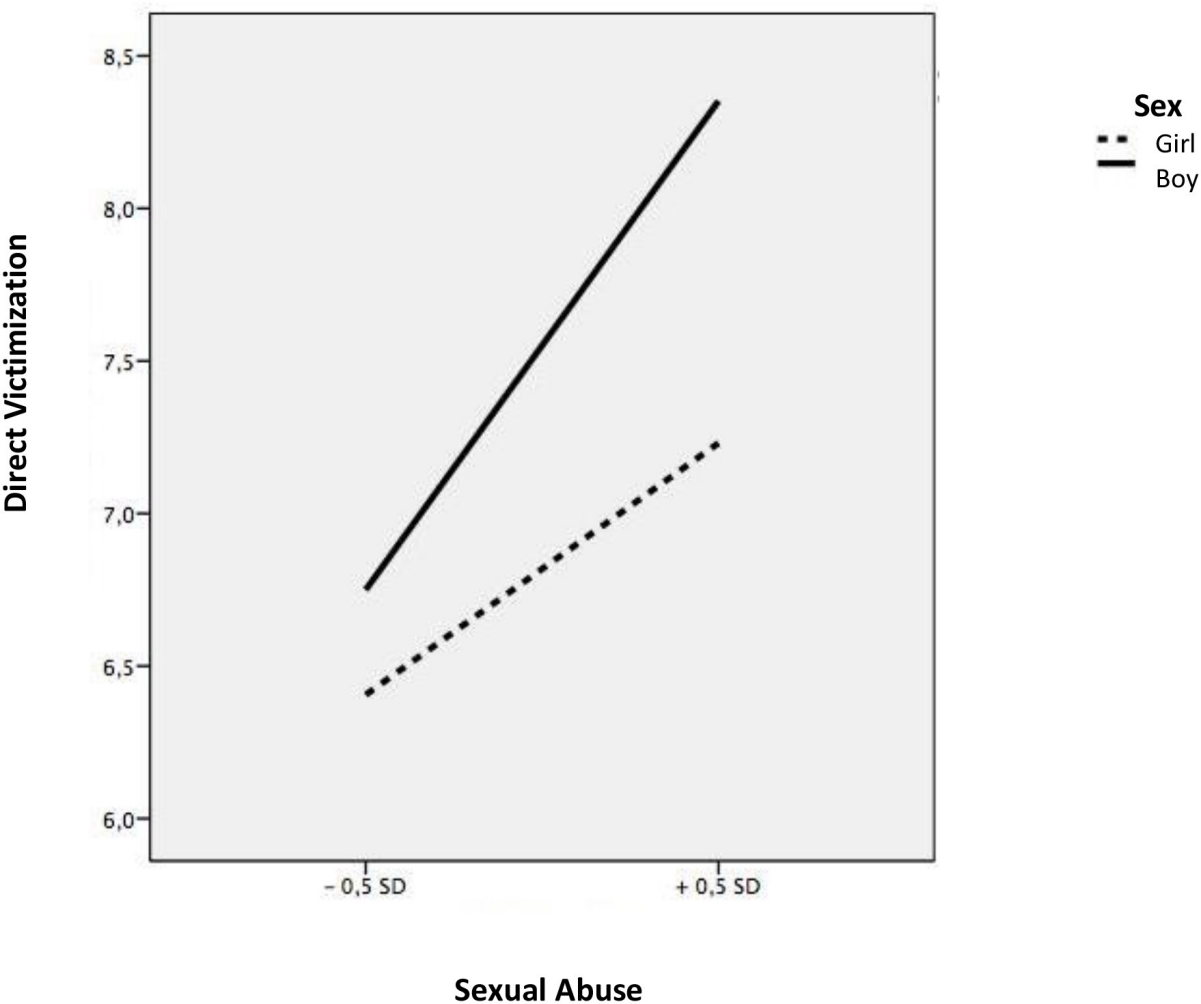
interaction between sexual abuse and sex in relation to direct victimization.

**Figure 3:**
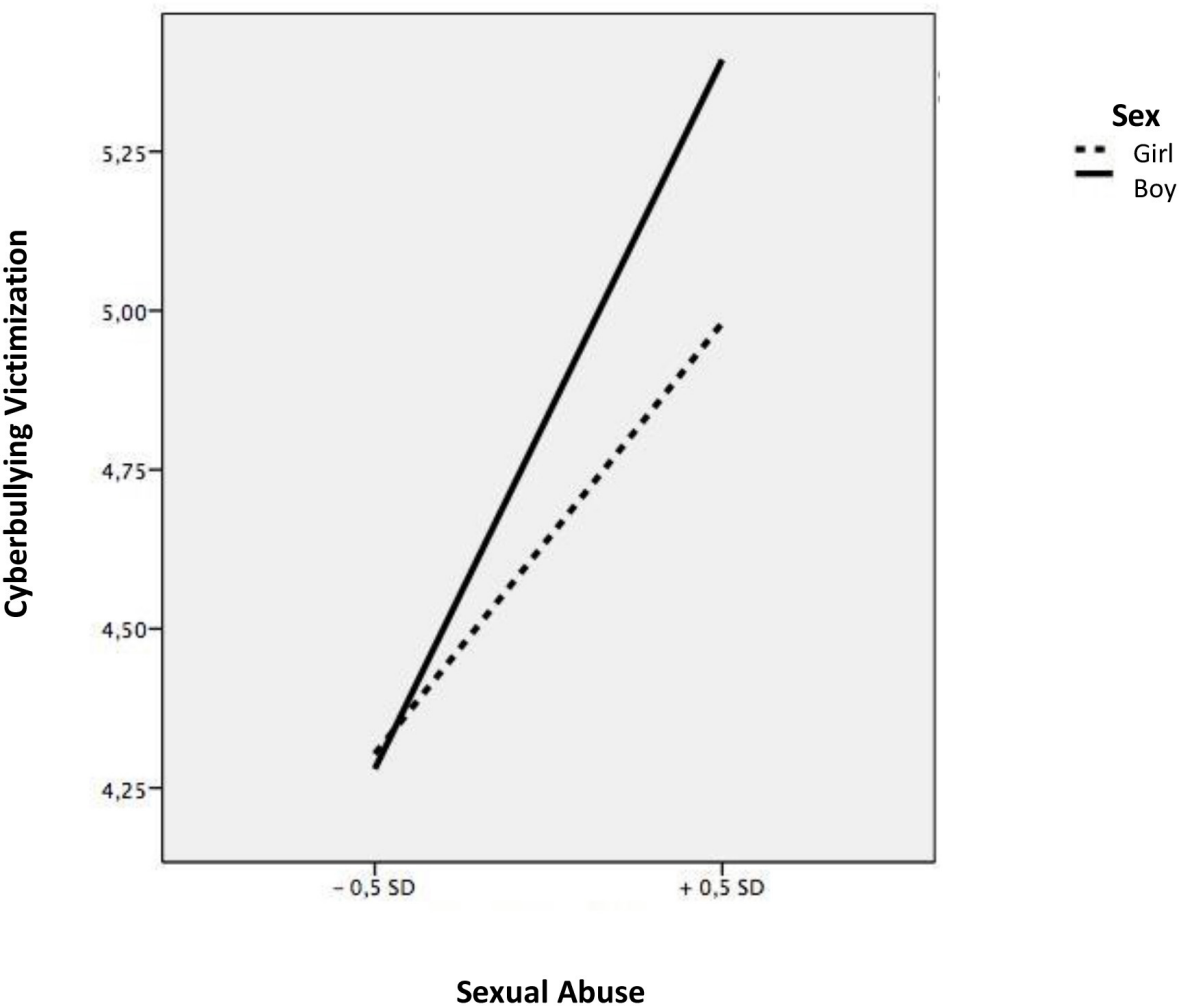
Interaction between sexual abuse and sex in relation to cyberbullying victimization.

The linear regression models showed that students who suffered emotional abuse within the family context had higher levels of indirect victimization (*b* = 0.48, *t* = 6.75, *p* < 0.001), direct victimization (*b* = 0.47, *t* = 4.89, *p* < 0.001) and cyberbullying (*b* = 0.85, *t* = 5.45, *p* < 0.001), while students suffering from physical abuse had higher levels of direct victimization (*b* = 0.29, *t* = 3.28, *p* < 0.001) (see Table 3). Next, we explored the moderating effect of sex and educational level. Sex showed an inter-rating effect, on the relationship of sexual abuse and the three types of school violence, indirect victimization (*b* = 0.19, *t* = 3.11, *p* < 0.003), direct victimization (continuous *b* =0.34, *t* = 4.24, *p* < 0.001), and cyberbullying (*b* = 0.45, *t* = 3.74, *p* < 0.001), while no significant associations were found in girls. To analyze these moderations in more detail, we calculated the simple slopes and the corresponding graphs according to the instructions provided by Aiken and West (1991). Sexual abuse was positively related to indirect victimization; however, this relationship was stronger for boy victims (b = 0.58, *t* = 5.14, *p* < 0.001) (Figure 1). Likewise, sexual abuse was shown to be positively associated with direct victimization; however, this relationship was stronger in the case of boys (b = 0.59, *t* = 9.10, *p* < 0.001), compared to girls (b = 0.24, *t* = 6.11, *p* < 0.001) (Figure 2). Finally, sexual abuse was shown to positively relate to cyberbullying more acutely in the case of boys (b = 0.39, *t* = 9.78, *p* < 0.001) compared to girls (b = 0.17, *t* = 7.23, *p* < 0.001) (Figure 3).

**Table 3:** Association of specific forms of child abuse and neglect with subtypes of peer victimization (*N* = 2.415).

	**Indirect victimization**		**Direct victimization**		**Cyberbullying**	
	***B***	**(SE)**	***B***	**(SE)**	***B***	**(SE)**
(Constant)	−0.36	(0.06)^**^	−1.32	(0.10)^***^	−2.67	(0.18)^***^
Sex (male = 1)	0.15	(0.06)	0.77	(0.09)^***^	0.16	(0.14)
Education (secondary = 1)	−0.28	(0.06)^***^	−0.31	(0.09)^**^	0.29	(0.15)
Emotional abuse	0.48	(0.07)^***^	0.47	(0.09)^***^	0.85	(0.15)^***^
Physical abuse	0.17	(0.07)	0.29	(0.08)^**^	0.13	(0.14)
Sexual abuse	0.09	(0.06)	−0.08	(0.08)	−0.10	(0.14)
Emotional neglect	0.01	(0.06)	0.05	(0.08)	0.22	(0.14)
Physical neglect	0.09	(0.06)	0.10	(0.08)	−0.16	(0.15)
Sex × emotional abuse						
Sex × physical abuse						
Sex × sexual abuse	0.19	(0.06)^*^	0.34	(0.08)^***^	0.45	(0.12)^***^
Sex × emotional neglect						
Sex × physical neglect						
Education × physical abuse						
Education × emotional abuse						
Education × sexual abuse	0.19	(0.07)^**^			0.34	(0.14)^†^
Education × physical neglect						
Education × emotional neglect						

*The Bonferroni correction was applied*.

† *p < 0.006*;

* *p < 0.003*;

** *p < 0.0006 (round to 0.001)*;

*** *p < 0.00005 (round to 0.001)*.

**Figure 4:**
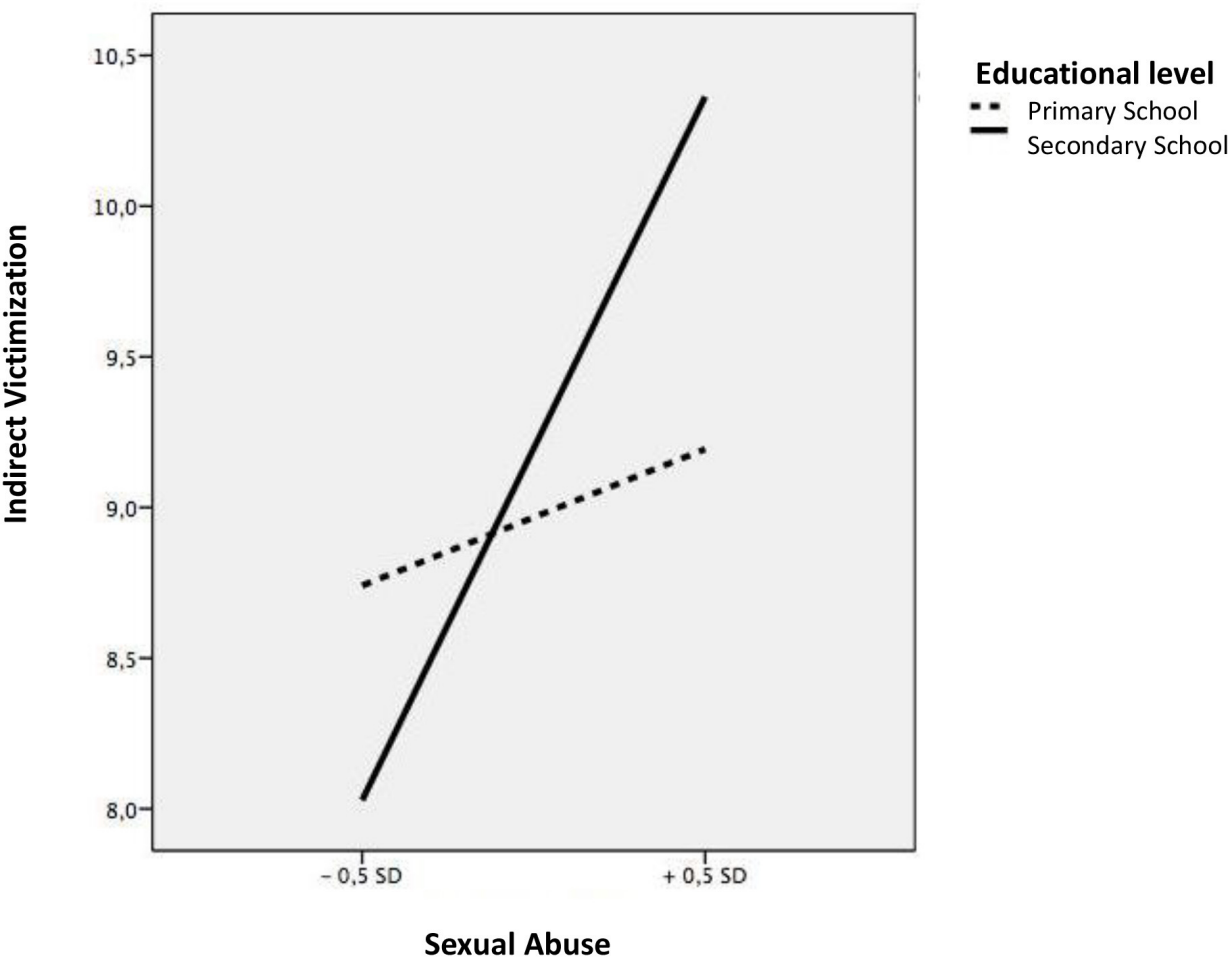
Interaction between sexual abuse and educational level in relation to indirect victimization.

**Figure 5:**
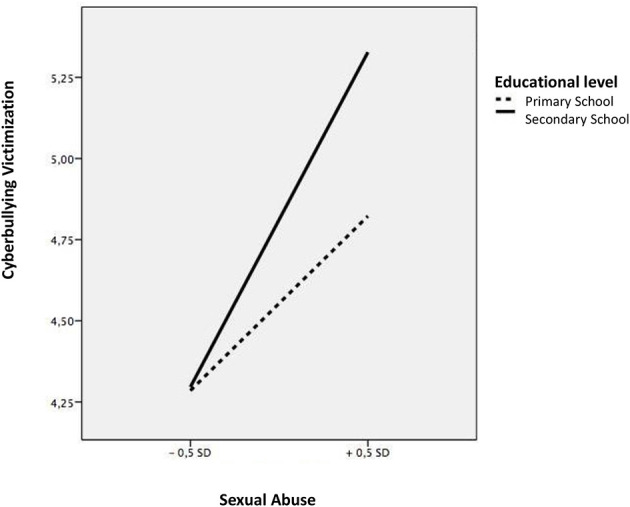
Interaction between sexual abuse and educational level in relation to cyberbullying victimization.

Then, we analyzed the moderating effect of educational level on the family maltreatment-peer victimization link. Sexual abuse was shown as one of the most relevant variables. Educational level showed an interacting effect on the relationship of sexual abuse with an indirect victimization relationship (*b* = 0.19, *t* = 2.58, *p* < 0.00). A more detailed analysis of this moderation indicated that this relationship between sexual abuse and indirect victimization was more accentuated during the secondary education stage (b = 0.57, *t* = 8.31, *p* < 0.001) compared to during the primary education stage (b = 0.08, *t* = 0.75, *p* < 0.001) (Figure 4). Educational level also showed an interacting effect on the association of sexual abuse with cyberbullying (b = 0.34, *t* = 2.35, *p* < 0.05). An analysis of this two-way interaction indicated that this relationship was more accentuated in secondary school (b = 0.27, *t* = 11.32, *p* < 0.001) than in the primary school (b = 0.12, *t* = 3.34, *p* < 0.001) (Figure 5).

## Discussion

In this large, cross-sectional study, we found that direct forms of maltreatment within the family, such as emotional or physical abuse, were positively associated with peer victimization in children and adolescents. Sex was found to moderate the association of sexual abuse with some forms of peer victimization; a significant association between sexual abuse and all subtypes of peer victimization was found only in boys. Our findings are along the lines of those of previous researchers who have reported a positive association between child maltreatment and peer rejection or victimization (Bolger and Patterson, 2001; Dussich and Maekoya, 2007; Kim and Cicchetti, 2010). This association can be understood in the context of ecological-transactional models (Cicchetti and Lynch, 1993), which assume that individual development is shaped by the multiple interactions and transactions of risk and protective factors between nested levels of influence (family, school, peers, media) (Cicchetti and Rizley, 1981). Within this conceptual framework, children and adolescents who are exposed to violent family environments may assume a role of victim and adopt the same role at school, leading to a transaction of the vulnerability to be victimized in the school environment. Children and adolescents who experience maltreatment by their primary caregivers have been found to show aggressive behavior outside the family context, particularly in peer relations at various academic levels (Widom, 1989; Espelage and Swearer, 2003), leading to increased conflicts with their peers and peer rejection (Kim and Cicchetti, 2010; Petersen et al., 2014). Child maltreatment is also associated with emotional maladjustment, internalizing and externalizing symptoms, and social withdrawal (Bolger and Patterson, 2001; Kim and Cicchetti, 2010), which might mediate its association with bullying (Liu et al., 2009; Hong et al., 2012). These emotional and behavioral patterns might lead to peers identifying a child as different, thus provoking peer rejection and conflicts. Such patterns have been consistently associated with an increased risk for experiencing peer victimization (Cook et al., 2010). There is evidence that family support and responsiveness to reports of victimization on bullying can promote child resilient behaviors and favor an earlier cessation of bullying experiences (Bowes et al., 2010). The lack of a supporting environment in families where abuse or neglect is present might reduce the reporting of bullying and lead to children's adoption of inefficient attitudes toward bullying, thereby perpetuating such situations.

Our results also suggest that there might be a differential effect of some forms of intrafamilial maltreatment and peer victimization. This would provide some additional support for the specificity of certain forms of abuse on psychosocial development and mental health outcomes (Teicher and Samson, 2016), although this is still a controversial issue. In our study, only forms of child maltreatment where the primary caregivers adopted a direct aggressive role (physical, emotional, or sexual abuse) were significantly associated with risk of peer victimization, while association was weaker for emotional or physical neglect. This is consistent with previous researchers' reporting that physical and emotional abuse within the family context is associated with increased peer victimization (Duncan, 1999) and perpetration (Shields and Cicchetti, 2001). This is possibly the consequence of aggressive rearing styles and active forms of maltreatment inflicting greater psychological damage on the child as compared with indirect forms of maltreatment, such as neglectful rearing styles. Children experiencing active aggression at home are more likely to repeat patterns of violence and victimization in other contexts, continuing the cycle of violence (Widom, 1989). Active forms of maltreatment and their associated negative parenting styles, such as authoritarian and overcontrolling (Baldry and Farrington, 1998; Ladd and Ladd, 1998), can also inhibit positive child behavior and lead to withdrawal and increased peer rejection.

We found that the association between sexual abuse and peer victimization was moderated by sex and educational level. Boys exposed to sexual abuse showed higher levels of peer victimization than did girls, and this association was constant for the three subtypes of bullying. In addition, a positive association of sexual abuse with peer victimization was found only in adolescents. Sexual abuse has been found to be associated with bullying perpetration (Shields and Cicchetti, 2001) and with peer victimization (Duncan, 1999; Turner et al., 2010) in children and in early adolescents. In a sample of college students discussing their experiences during childhood, Duncan (1999) found that 29% of bullying victims compared to 9% of those who had been sexually assaulted were not victims of bullying. Furthermore, Turner et al. (2010) found that 50% of children who suffered from sexual victimization also reported being poly-victimized. These earlier pieces of research did not detect a moderating effect of sex or age on the effect of sexual abuse. We posit that of those suffering from sexual abuse, boys are more likely than girls to show more overt manifestations of unwell being, such as externalizing symptoms or aggressive behavior, leading to more frequent conflicts with peers.

These results highlight the importance of understanding difficulties and conflicts in a peer context in direct relation to difficulties in the family context and not as isolated systems. Both contexts need to be incorporated and integrated more prominently, for example when developing anti-bullying programs. Peer victimization and family abuse share underlying characteristics, both of which are based on an imbalance of power. It is necessary to deepen the study of how this imbalance and the emotional learning received in the primary family group are related and possibly transferred to other contexts. This study highlights the importance of finding new ways to understand the different types of abuse, the imprint and the mismatch produced by an active and direct style vs. a more indirect style. There is a need to deepen the study of more specific characteristics of family abuse, such as its intensity, correlations with poly-victimization, and its relationship with social performance in the context of peers or other environments. The early detection of student difficulties in social adaptation within the classroom could facilitate the detection and correction of difficulties in the family environment.

### Strengths and Limitations

This study is subject to several limitations. First, this was a cross-sectional study, which does not allow for inferring the direction of the association between child abuse and peer victimization. Even if from a theoretical perspective we expected child maltreatment to be associated positively with an increased risk of experiencing peer victimization, a bidirectional effect remains possible, with children and adolescents experiencing peer victimization at school, showing greater behavioral and emotional disturbances at home, which might cause some forms of abuse within the family. Second, we relied on self-report measures of childhood abuse and peer victimization. Even if self-report measures are very common in bullying research and are usually considered to be valid and reliable (Ladd and Kochenderfer-Ladd, 2002), significant inconsistencies still remain with respect to bullying definitions and measurement strategies currently used in studies (Vivolo-Kantor et al., 2014). Previous research has shown the potential added value of complementary assessment methods such as those based *via* peer nomination and sociometric assessment (Coie et al., 1982; Bouman et al., 2012), or those instruments that incorporate an analysis of the group structure in which bullying occurs (Martín Babarro, 2014).

Third, we used a scale for assessing child abuse that was not validated in the Mexican population. Fourth, our results should be appraised in the social, cultural, and economic context of the country where the study was performed. Although there is no accurate data on child maltreatment and peer victimization at school, Mexico ranks first in child maltreatment and bullying among the nations that belong to the Organization for Economic Co-operation and Development (OECD, 2011). According to UNICEF (2009), between 55 and 62% of high school students reported that they had experienced child maltreatment at some point in their lives, 90% of the school population has suffered humiliation and insults, and at least two thirds reported receiving at least one physical assault. It might be difficult to extrapolate this study's results to populations with social and economic characteristics different from those of Mexico. Although the intention was to sample a diverse pool of Mexican children and public-school adolescent students with the same socioeconomic level, there is a huge socio-economic gap between public and private schools. So, our results cannot be generalized to all educational contexts. Future research should compare these findings with different socioeconomic taxonomies, such as from private institutions or schools in different countries.

Nevertheless, this study adds a cross-cultural perspective to the issue, by providing further support for the interrelationship between both forms of child victimization that have previously been reported in low-income subpopulations of high-income countries (Bolger and Patterson, 2001; Kim and Cicchetti, 2010), in a country of lower income than previous studies. Fifth, the design of the study did not allow for measuring clinical variables such as depressive or anxiety symptoms, emotional regulation, or social skills, which seem to be relevant aspects in the study of child abuse and peer victimization (Schwartz et al., 1997; Hong et al., 2012). Sixth, we did not control for potential confounding variables that might predispose both child abuse and bullying such as socio-economic status.

Despite these limitations, this study provides further evidence for the presence of risk of multi-victimization in children exposed to violence in different contexts during their development, based on a large sample of children and adolescents. This is especially relevant in light of increasing evidence of biological changes in children exposed to violence and trauma, rendering them more sensitive to later stressful situations and leading to maladjustment and an increased risk for adverse mental and medical outcomes (Teicher and Samson, 2016). Our results indicate that direct forms of victimization within the family such as emotional or physical abuse are positively associated with the likelihood of peer victimization at school, which suggests a differential effect of some forms of child maltreatment on the risk of bullying. In the case of sexual abuse, this association seems to be especially relevant for males and might become more apparent during adolescence.

Future studies combining self-report measures with other sources of information and using a longitudinal design can provide relevant information on the issue. These studies could (1) provide valuable information on the effect of the timing, degree of severity and chronicity of abuse experiences during childhood on the risk of peer victimization (Bolger et al., 1998); (2) test the directionality of the associations; and (3) explore whether the associations found in this study might be subject to change over time. Prevention and early intervention strategies should aim at identifying and providing support for high-risk families and young people at risk for multi-victimization. Approaches to tackling bullying and promoting resilience should also take family, individual risk, and protective factors into account, including previous victimization within the family context. Such approaches could help reduce the long-term negative consequences of both child maltreatment and peer victimization.

## Data Availability Statement

The raw data supporting the conclusions of this article will be made available by the authors, without undue reservation.

## Ethics Statement

Ethical review and approval was not required for the study on human participants in accordance with the local legislation and institutional requirements. Written informed consent to participate in this study was provided by the participants' legal guardian/next of kin.

## Author Contributions

JF-S was involved in planning and supervise the work. JM-B directed the project and did the analysis of the results. MT designed and implemented the research with NGO and Ministry of Education in Mexico. RA-C and MT contributed by preparing the online questionnaire and recruiting the participating schools. CD-C and LP-B contributed to the writing and review of the manuscript with input from all authors.

## Conflict of Interest

CD-C has received grant support from Instituto de Salud Carlos III, Spanish Ministry of Science and Innovation (JR19/00024, PI17/00471, PI20/00721), co-financed by ERDF funds from the European Commission, “A way of making Europe”, and honoraria from Sanofi and Exeltis. The remaining authors declare that the research was conducted in the absence of any commercial or financial relationships that could be construed as a potential conflict of interest.

## Publisher's Note

All claims expressed in this article are solely those of the authors and do not necessarily represent those of their affiliated organizations, or those of the publisher, the editors and the reviewers. Any product that may be evaluated in this article, or claim that may be made by its manufacturer, is not guaranteed or endorsed by the publisher.
